# Electronic Medical Record Search Engine (EMERSE): An Information Retrieval Tool for Supporting Cancer Research

**DOI:** 10.1200/CCI.19.00134

**Published:** 2020-05-15

**Authors:** David A. Hanauer, Jill S. Barnholtz-Sloan, Mark F. Beno, Guilherme Del Fiol, Eric B. Durbin, Oksana Gologorskaya, Daniel Harris, Brett Harnett, Kensaku Kawamoto, Benjamin May, Eric Meeks, Emily Pfaff, Janie Weiss, Kai Zheng

**Affiliations:** ^1^ Department of Pediatrics, University of Michigan Medical School, Ann Arbor, MI; ^2^ Case Western Reserve University School of Medicine, Cleveland, OH; ^3^ University Hospitals of Cleveland, Cleveland, OH; ^4^ Cleveland Institute for Computational Biology, Cleveland, OH; ^5^ Department of Biomedical Informatics, University of Utah, Salt Lake City, UT; ^6^ Markey Cancer Center, UK HealthCare, Lexington, KY; ^7^ Division of Biomedical Informatics, University of Kentucky, Lexington, KY; ^8^ Clinical and Translational Science Institute, University of California San Francisco, San Francisco, CA; ^9^ Department of Biomedical Informatics, University of Cincinnati College of Medicine, Cincinnati, OH; ^10^ Herbert Irving Comprehensive Cancer Center, Columbia University, New York, NY; ^11^ North Carolina Translational and Clinical Sciences Institute, University of North Carolina School of Medicine, Chapel Hill, NC; ^12^ Department of Informatics, University of California, Irvine, CA

## Abstract

**PURPOSE:**

The Electronic Medical Record Search Engine (EMERSE) is a software tool built to aid research spanning cohort discovery, population health, and data abstraction for clinical trials. EMERSE is now live at three academic medical centers, with additional sites currently working on implementation. In this report, we describe how EMERSE has been used to support cancer research based on a variety of metrics.

**METHODS:**

We identified peer-reviewed publications that used EMERSE through online searches as well as through direct e-mails to users based on audit logs. These logs were also used to summarize use at each of the three sites. Search terms for two of the sites were characterized using the natural language processing tool MetaMap to determine to which semantic types the terms could be mapped.

**RESULTS:**

We identified a total of 326 peer-reviewed publications that used EMERSE through August 2019, although this is likely an underestimation of the true total based on the use log analysis. Oncology-related research comprised nearly one third (n = 105; 32.2%) of all research output. The use logs showed that EMERSE had been used by multiple people at each site (nearly 3,500 across all three) who had collectively logged into the system > 100,000 times. Many user-entered search queries could not be mapped to a semantic type, but the most common semantic type for terms that did match was “disease or syndrome,” followed by “pharmacologic substance.”

**CONCLUSION:**

EMERSE has been shown to be a valuable tool for supporting cancer research. It has been successfully deployed at other sites, despite some implementation challenges unique to each deployment environment.

## INTRODUCTION

The vast volume of clinical data captured within electronic health records (EHRs) has the potential to catalyze biomedical research. However, for all the benefits of EHRs, persistent challenges remain in leveraging EHR data for cancer research. This is because a substantial number (up to 80% by some estimates)^1^ of the clinical details are captured in unstructured free-text notes and are therefore difficult to extract and convert to a computable form.^2^

**CONTEXT**

**Key Objective**
To demonstrate the utility of an information retrieval system, the Electronic Medical Record Search Engine (EMERSE), in the context of supporting cancer research.
**Knowledge Generated**
An analysis of audit logs and peer-reviewed publications demonstrated that EMERSE is being used to support cancer research for a broad array of research projects and tasks, ranging from cohort identification to data abstraction for elements that may not be found in a structured form. Users are searching for a wide variety of concepts, including “pharmacologic substance,” “neoplastic process,” and “sign or symptom.”
**Relevance**
Information retrieval systems such as EMERSE have the potential to be powerful and easy-to-use software tools for supporting cancer research. EMERSE is available at no cost and has been successfully implemented at multiple medical centers, so it is a viable option for sites seeking to provide additional software tools for supporting cancer research.

Ignoring the free text in EHRs can be problematic.^3^ For example, symptomatic data are often recorded exclusively in the free text.^4^ One study found that free text from EHRs was required for resolving nearly 60% of eligibility criteria for a chronic lymphocytic leukemia clinical trial and almost 80% of eligibility criteria for a prostate cancer trial.^5^ Another such study listed 10 data elements derived from the free text related to bone marrow biopsy findings, including biopsy blast counts, biopsy cellularity, fibrosis grade, and aspirate cellularity.^6^ A study about engraftment syndrome after allogeneic hematopoietic cell transplantation used concepts found in the free text, such as engraftment failure, stool output, lymphocyte recovery, cytokine storm, disorientation, capillary leak, effusions, fevers, and rashes.^7^ Furthermore, the accuracy of the readily accessible structured data from EHRs may be low in some cases.^8^ For example, one study found that up to 20% of patients at one medical center had a medication listed in their unstructured data that was not in the structured medication list.^9^ Another study of cancer staging found that nearly 84% of patients had conflicting statements about staging in their records, necessitating an algorithm to infer the most likely staging for each patient.^10^

To help the research community use the free text in EHRs, substantial resources have been devoted to develop natural language processing (NLP) tools. NLP remains promising for oncology research,^11^ but widespread use remains limited. The quality of NLP results have been mixed, with some acknowledging the complexity and “inherent difficulty of natural language processing in this domain.”^6(p330-331)^ This complexity results from a variety of factors, including understanding temporal relationships, ambiguous abbreviations, and anaphoric references. Other challenges include issues of replicability across algorithms and institutions^12^ and the need for large manually annotated data sets for new use cases,^11^ especially because these systems perform best when tailored to a specific task or domain.^13^ The lack of available experts to architect and deploy NLP systems is also a limiting factor.

To address the immediate needs of the cancer research community, members of which often lack the resources, time, and access to NLP experts, we developed a simpler approach using information retrieval for concept identification in free text. The Electronic Medical Record Search Engine (EMERSE) is a general-purpose term-searching system tailored to the needs of the medical research community to help researchers quickly find information buried in EHR free text. In general, information retrieval is like search engines such as Google that help people find information quickly, but it does not attempt to code the data, the latter of which falls within the domain of NLP. General familiarity with tools such as Google is thus an advantage. EMERSE uses an index of terms coupled with the capacity for query expansion using locally customized or standardized terminologies.

Rather than an example of an artificial intelligence system, EMERSE is more like an augmented intelligence system, wherein the software helps a person perform his or her work more efficiently but does not completely remove that person from the workflow. With EMERSE, the person is needed for the complex task of making sense of nuanced prose, a task that remains formidable for machines.^14^ EMERSE has been in use at the University of Michigan for 15 years and has supported a wide variety of clinical research, including oncology research. EMERSE is being implemented at other academic medical centers. Our report covers details about the system, including metrics based on use logs and publications, an analysis of search terms entered, and ongoing development work supported by the National Cancer Institute Informatics Technology for Cancer Research program.

## METHODS

### System Description

EMERSE is a Web-based application that provides an easy-to-use interface for either (1) identifying a cohort among all patients in the EHR or identifying concepts within the clinical unstructured notes of an existing defined patient cohort. EMERSE indexes free-text data from EHR notes, with additional metadata related to the notes (eg, date, clinical service, note type). The software is based on Apache Solr (an open-source search engine), but a substantial user interface has been built to provide study management features, visualization of results, and a query expansion feature.

**FIG 1.**
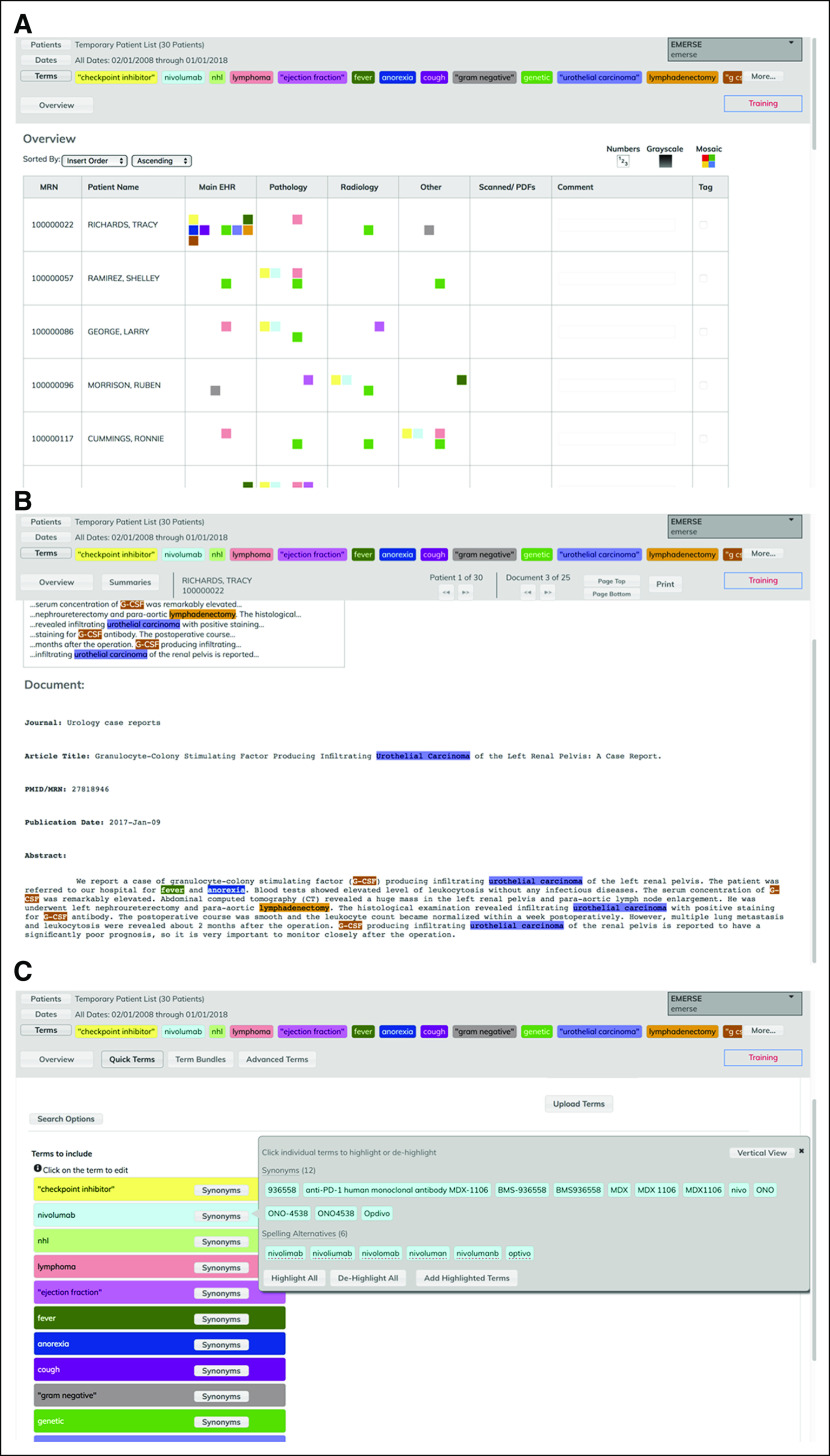
Screenshots of some basic features within Electronic Medical Record Search Engine (EMERSE). (A) Overview in which each row represents a patient in a list, and columns represent document sources. The colors in each cell represent terms for each patient and source that appear in that patient’s documents. The colors are associated with the colors of the highlighted search terms, shown across the navigation panel at the top. (B) Example of a specific note (in this case, a PubMed abstract; names and medical record numbers are fake), with the terms still highlighted in the note. (C) Term expansion feature, with additional synonyms for nivolumab shown.

**FIG 2.**
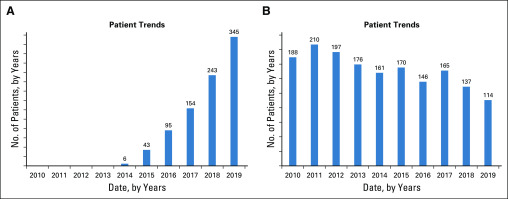
Examples of the graphs available within the Electronic Medical Record Search Engine (EMERSE) trends feature. The graphs have been redrawn from the original screenshots for clarity within this publication. The graphs show the number of distinct patients per year with at least one note in the electronic health record mentioning the search term of interest, which can be useful for looking at patient trends over time. (A) Rapid increase in the mention of checkpoint inhibitor. (B) Gradual decrease in the mention of radical mastectomy, ignoring notes that mention modified radical mastectomy (query: “radical mastectomy” NOT “modified radical mastectomy”). Note that 2019 data are through mid December.

Technical details about EMERSE can be found in a prior publication.^15^ EMERSE maintains detailed audit logs for all user sessions. Figure 1 contains several screens from EMERSE showing various general functions of the system. A recently added feature visualizes trends over time based on the search terms of interest (Fig 2). Although EMERSE is intended to be a self-service tool, system support is expected to be managed centrally by groups such as operational informatics teams. EMERSE is available at no cost, including source code, but sites are required to contact the University of Michigan to obtain the software. Additional details about EMERSE, including documentation and explainer videos, can be found on the EMERSE project Web site.^16^

EMERSE is currently in use at three academic medical centers: University of Michigan, University of North Carolina at Chapel Hill, and University of Cincinnati (Table 1). Other sites are currently at various stages in their implementation, including Case Western Reserve University (CWRU)/University Hospitals of Cleveland, Columbia University, University of Kentucky, University of Utah, and University of California San Francisco. CWRU has implemented a version of EMERSE using data extracted from the MIMIC-III project^17^ and plans to use EMERSE in a pilot program for training medical students about research software and as part of its health informatics training program.

**TABLE 1.**
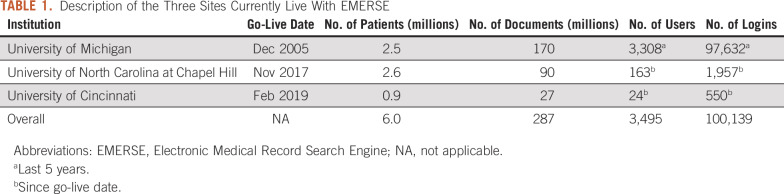
Description of the Three Sites Currently Live With EMERSE

### Publication Data

Peer-reviewed publications using EMERSE were identified via manual searches for “EMERSE” or “electronic medical record search engine” in both PubMed and Google Scholar. Searches were conducted between August and September 2019. Each article identified was reviewed to confirm EMERSE use. To identify additional peer-reviewed publications without mention or citation of EMERSE, all principal investigators at the University of Michigan who had used EMERSE for research within the prior 5-year period (n = 600) were sent an e-mail in July/August 2019 to inquire about the use of EMERSE for their work and what publications arose from that use. The e-mail contained personalized audit logs to remind them about the use. A follow-up e-mail to nonresponders was sent in early September 2019. For all articles identified, the titles and abstracts were read to determine if they were cancer related.

To characterize how EMERSE was used to support various research initiatives, 47 recent cancer-related peer-reviewed publications published within the last 2 years were reviewed. Among these, 11 were summarized with respect to their descriptions of how EMERSE was used. These 11 articles were selected to showcase a diversity of use cases, were from a variety of research teams from different disciplines, and had enough details described in their methods sections to understand the contribution of EMERSE.

### Audit Log Analysis

Use logs were extracted to characterize the total number of users and the number of EMERSE logins over the past 5 years (September 2014 through August 2019; shorter timeframes for the two sites that recently adopted the system). The search terms (ie, search queries) entered within this timeframe were also extracted. The NLP tool MetaMap^18^ was used to process the search terms from two of the sites (University of Michigan and University of Cincinnati; University of North Carolina did not provide its terms). For this analysis, the “-a -N” flags were used. The “-a” flag enables the use of variants of acronyms and abbreviations, and the “-N” flag modifies how the output is displayed. Prior studies have shown that MetaMap can perform comparably to other NLP tools, such as cTAKES.^19^

MetaMap processed each search term to determine if MetaMap could map the query to a concept unique identifier (CUI) within the Unified Medical Language System (UMLS)^20^ and, if the concept could be identified, to what semantic type it belonged. Because MetaMap outputs a list of potential CUI candidates, only the top-scoring candidate was selected. For ties among top-scoring candidates, only the first was selected. The results across the two sites were merged, and the relative frequencies of the top 20 most common UMLS semantic types were visualized using RAWGraphs.^21^

## RESULTS

A total of 222 peer-reviewed publications were identified through manual searches using PubMed and Google Scholar through September 19, 2019. For the e-mail survey that was conducted to gain additional data about publications, 337 (56.2%) of the 600 principal investigators responded, revealing an additional 105 peer-reviewed publications that did not cite or mention EMERSE, bringing the total number of publications to 326. Of the 326 publications, 105 (32.2%) were oncology related. An additional 285 studies were still in progress, with potential publications coming at a later date. The current list of known peer-reviewed publications can be found on the EMERSE project Web site.^16^ Summaries of how EMERSE was used for 11 selected oncology-related articles are provided in Table 2. The use of EMERSE varied from cohort identification to various types of data abstraction.

**TABLE 2.**
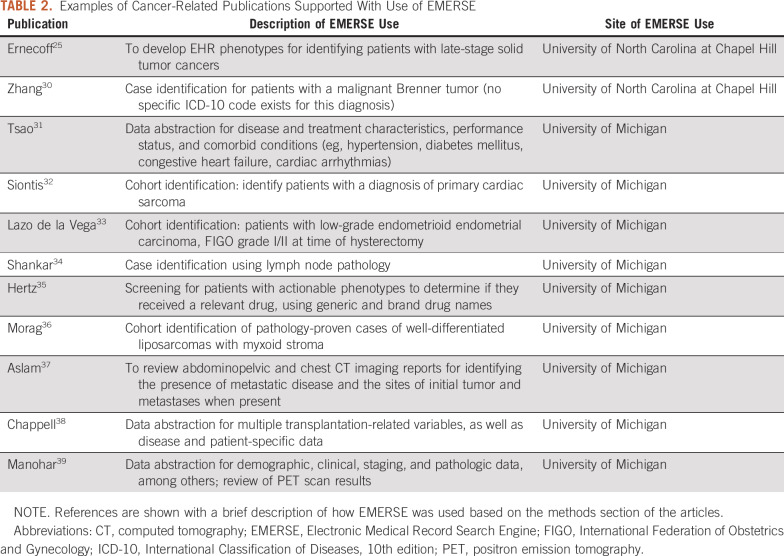
Examples of Cancer-Related Publications Supported With Use of EMERSE

The audit logs revealed substantial use of EMERSE for cancer-related work that did not acknowledge EMERSE use within publications. This included multisite clinical trials where EMERSE was used at a single site (University of Michigan). These publications could be identified via unique data, such as National Clinical Trial numbers, which were sometimes mentioned in the publications. Examples include one study that used EMERSE for 31 sessions, with a total session time of 13 hours (ClinicalTrials.gov identifier: NCT01865747),^22^ another that used EMERSE for 58 sessions and 26 hours (ClinicalTrials.gov identifier: NCT01576172),^23^ and a third that used EMERSE for 398 user sessions and 166 hours (ClinicalTrials.gov identifier: NCT01633372).^24^

Other oncology-related research initiatives have used EMERSE, even though it is not possible to link the use back to specific studies. For example, the Michigan Medicine Oncology Clinical Trials Support Unit has an umbrella institutional review board application for which it accesses EMERSE but does not link use to a specific study. That unit logged into EMERSE 917 times for 388 hours of use on the system between December 2014 and July 2019. Additionally, the Bone Marrow Transplant research group uses EMERSE for tracking long-term outcomes and used EMERSE for 2,452 sessions and 1,106 hours between July 2014 and July 2019. The high number of logins per study is common for research that involves frequent patient monitoring or identification of adverse events. Additional use statistics are listed in Table 1.

**FIG 3.**
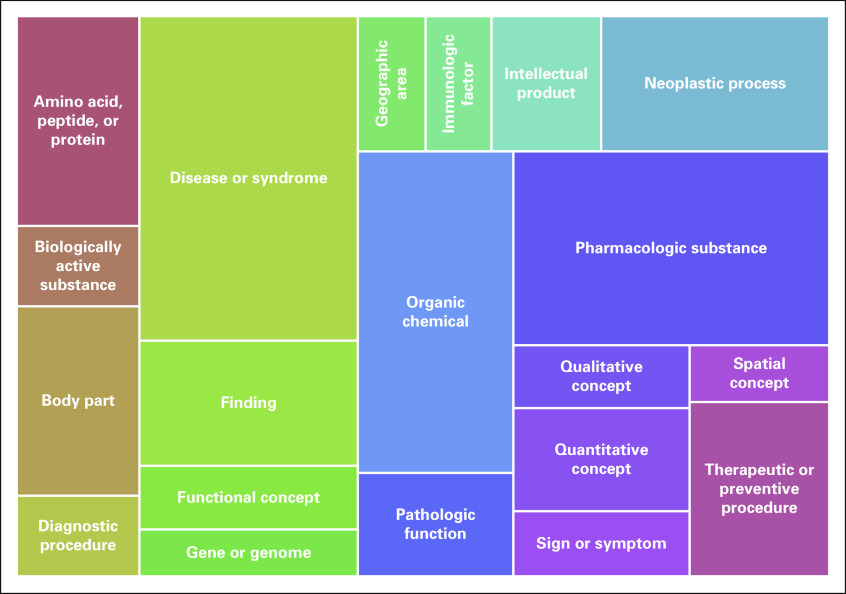
Tree map showing the relative frequency of the top 20 most common semantic types based on search terms entered, with data combined from the University of Michigan and University of Cincinnati. These 20 semantic types together represent 74.3% of all of the concept unique identifiers identified by applying MetaMap to the search terms.

Details about the analysis of search terms using MetaMap are listed in Table 3. A large number of terms (University of Michigan, 34.1%; University of Cincinnati, 55.9%) did not map to any CUI using MetaMap. Many of these nonmapping terms were misspellings (eg, “fludaribine,” “ifosphomide,” “pegasparaganase,” “tamoxafen”). However, of the terms that did not map from the University of Michigan data set, 2,342 (9.0%) were numbers in various forms representing medical record numbers, dates, international classification of disease (ICD) codes, and even pathology slide identifiers. In the University of Cincinnati data set 1,975 (68.6%) of the terms that did not map were numbers. The relative frequency of the 20 most common semantic types for the search terms is shown in Figure 3. “Disease or syndrome” was the most frequent semantic type (11.5%), followed by “pharmacologic substance” (10.0%).

**TABLE 3.**
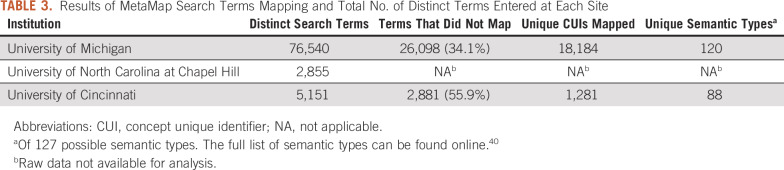
Results of MetaMap Search Terms Mapping and Total No. of Distinct Terms Entered at Each Site

## DISCUSSION

As shown by the audit logs, and as evidenced by numerous peer-reviewed publications (> 100 oncology related), EMERSE has proven to be a useful tool for supporting cancer research. Furthermore, EMERSE has been successfully deployed at three academic medical centers to date, including the University of North Carolina, with additional centers in process, leading to multiple peer-reviewed publications.^25^

Through several rounds of implementation work with other sites (several are still under way), we have learned a great deal about the complexities of enterprise-wide software implementation. We describe a few of the most important insights, provided as guidance for others who might be interested in implementing EMERSE or other centrally managed research tools.

Environments at each site are highly variable, including servers, storage, access to EHR documents, formats of these documents, and regulatory requirements. Although there is no cost per se for the software, the resources needed for implementation are not free. Competing priorities, institutional review board requirements, small teams, security reviews, and the need to obtain buy-in from leadership can delay implementation for months. There is no single solution to overcoming these challenges, but we have made efforts to reduce the burden on implementing sites, including providing installation and setup documentation, training materials for end users, and a messaging forum for technical teams.

Because EMERSE is meant to be user facing, preserving the original document formatting helps users understand the data in the notes. Modern EHRs, such as Epic, allow for documentation using rich text formatting, in which notes can be made with tables, line breaks, and other formatting (eg, bold-face text). However, the Epic analytics database, Clarity, almost universally stores a version of the notes stripped of all formatting. The University of North Carolina at Chapel Hill has avoided using Clarity and is using the live production database, Chronicles, instead.

The University of Utah, one of our partners, is working on a solution based on application program interfaces compliant with the Health Level Seven Fast Healthcare Interoperability Resources^26^ standard that should solve this challenge by extracting formatted notes in bulk. This approach is aligned with priorities of the US National Institutes of Health to “explore the use of the Fast Healthcare Interoperability Resources (FHIR) standard to capture, integrate, and exchange clinical data for research purposes and to enhance capabilities to share research data.”^27(p1)^ Other sites, such as University of Cincinnati, have used simple logic and regular expressions to rebuild functional formatting in the notes.

Contrary to when EMERSE was first developed and deployed, security considerations are becoming a top priority, as they are for any software that contains protected health information within a medical center. This focus on security requires substantial, ongoing resources for conducting repeated scans for vulnerabilities that exist in the underlying open-source components, as well as in the system configuration, code reviews, penetration testing, and other measures. This work adds to the development costs but is a necessary component that other sites are requiring before considering a deployment. The importance of software security, as well as local institutional policies, should not be underestimated.

Finally, demonstrating the value, effectiveness, and return on investment of software such as EMERSE remains challenging, especially if one considers peer-reviewed publications to be the gold standard of evidence. As demonstrated by the number of times the tool was used but never cited or mentioned, referencing software tools are not a top priority for many in the research community. However, this type of attribution is important to ensure future funding for software development teams, which can be expensive.

For the analysis of semantic types, it is worth noting that only a few of the semantic types identified are for data typically found in the structured section of EHRs (eg, diseases, pharmaceutical substances). Many of the other concepts are likely to be found only in the free-text notes. Furthermore, many of the terms entered by users were not mappable by the popular NLP tool MetaMap. This could be because of limitations of current NLP tools or because users of EMERSE are searching for concepts that do not have a matching CUI or semantic type within UMLS.

The performance of MetaMap in our case likely could have been improved by adding an additional preprocessing step wherein incorrectly spelled terms would be mapped back to their correct spellings. Even though signs and symptoms are almost exclusively noted in the narrative portion of the medical record, these did not represent the most frequent semantic type. However, this may be because our analysis was performed on a unique list of terms in the search logs, and there may be far fewer signs and symptoms than there are disease or drug names.

Additional work under way involves securely networking sites for obfuscated counts. This feature will be similar to other cohort discovery networks currently based on structured data, such as i2b2 ACT^28^ and PCORnet,^29^ but the novelty with the EMERSE-based network is the focus on free-text notes. This should be useful for finding rare cancer cases where structured data are not specific enough. For example, there is no specific code in the ICD (version 10) for endometrial stromal sarcoma, because the parent code C54.1 represents multiple types of endometrial neoplasms.

It is important to point out that EMERSE is not meant to be a replacement for NLP systems, and NLP will be a preferable option in certain use cases. For relatively small numbers of patients (eg, thousands) and where accuracy is important enough to warrant human review, EMERSE may be the tool of choice. In other situations, such as automatically coding data across hundreds of thousands or millions of patients, NLP may be a preferable option. There is no one-size-fits-all solution, and multiple tools can benefit the research enterprise.

In conclusion, EMERSE can be a valuable tool to support cancer research as well as other clinical domains. This is a simple-to-operate, self-service tool that is powerful, scalable, and generalizable across use cases, allowing for teams from various fields to increase their productivity and gain access to accurate patient data that normally would have required a manual approach for identification. In addition, it has many data security features. Successful implementation at other locations has demonstrated that EMERSE can be deployed and used outside its original site. Groups interested in adopting EMERSE can contact the EMERSE team at the University of Michigan for a working virtual machine for testing, demonstrations, advice, and other details.

## References

[1] Murdoch TB Detsky AS The inevitable application of big data to health care JAMA 309 1351 1352 2013 23549579 10.1001/jama.2013.393

[2] Polnaszek B Gilmore-Bykovskyi A Hovanes M et al Overcoming the challenges of unstructured data in multisite, electronic medical record-based abstraction Med Care 54 e65 e72 2016 27624585 10.1097/MLR.0000000000000108 PMC5024721

[3] Kharrazi H Anzaldi LJ Hernandez L et al The value of unstructured electronic health record data in geriatric syndrome case identification J Am Geriatr Soc 66 1499 1507 2018 29972595 10.1111/jgs.15411

[4] Hernandez-Boussard T Tamang S Blayney D et al New paradigms for patient-centered outcomes research in electronic medical records: An example of detecting urinary incontinence following prostatectomy EGEMS (Wash DC) 4 1231 2016 27347492 10.13063/2327-9214.1231 PMC4899050

[5] Raghavan P Chen JL Fosler-Lussier E et al How essential are unstructured clinical narratives and information fusion to clinical trial recruitment? AMIA Jt Summits Transl Sci Proc 2014 218 223 2014 PMC4333685 25717416

[6] Sholle E Krichevsky S Scandura J et al Lessons learned in the development of a computable phenotype for response in myeloproliferative neoplasms IEEE Int Conf Healthc Inform 2018 328 331 2018 31276120 10.1109/ICHI.2018.00045 PMC6608705

[7] Chang L Frame D Braun T et al Engraftment syndrome after allogeneic hematopoietic cell transplantation predicts poor outcomes Biol Blood Marrow Transplant 20 1407 1417 2014 24892262 10.1016/j.bbmt.2014.05.022 PMC4142041

[8] Birman-Deych E Waterman AD Yan Y et al Accuracy of ICD-9-CM codes for identifying cardiovascular and stroke risk factors Med Care 43 480 485 2005 15838413 10.1097/01.mlr.0000160417.39497.a9

[9] Walsh KE Marsolo KA Davis C et al Accuracy of the medication list in the electronic health record-implications for care, research, and improvement J Am Med Inform Assoc 25 909 912 2018 29771350 10.1093/jamia/ocy027 PMC7647042

[10] Warner JL Levy MA Neuss MN et al ReCAP: Feasibility and accuracy of extracting cancer stage information from narrative electronic health record data J Oncol Pract 12 157 158, e169-e7 2016 26306621 10.1200/JOP.2015.004622

[11] Savova GK Danciu I Alamudun F et al Use of natural language processing to extract clinical cancer phenotypes from electronic medical records Cancer Res 79 5463 5470 2019 31395609 10.1158/0008-5472.CAN-19-0579 PMC7227798

[12] Carrell DS Schoen RE Leffler DA et al Challenges in adapting existing clinical natural language processing systems to multiple, diverse health care settings J Am Med Inform Assoc 24 986 991 2017 28419261 10.1093/jamia/ocx039 PMC6080843

[13] Kreimeyer K Foster M Pandey A et al Natural language processing systems for capturing and standardizing unstructured clinical information: A systematic review J Biomed Inform 73 14 29 2017 28729030 10.1016/j.jbi.2017.07.012 PMC6864736

[14] Gorski D: IBM Watson: Not living up to hype as a tool to fight cancer? https://scienceblogs.com/insolence/2017/09/18/ibm-watson-not-living-up-to-hype-as-a-tool-to-fight-cancer

[15] Hanauer DA Mei Q Law J et al Supporting information retrieval from electronic health records: A report of University of Michigan’s nine-year experience in developing and using the Electronic Medical Record Search Engine (EMERSE) J Biomed Inform 55 290 300 2015 25979153 10.1016/j.jbi.2015.05.003 PMC4527540

[16] EMERSE: Electronic Medical Record Search Engine. http://project-emerse.org

[17] Johnson AEW Pollard TJ Shen L et al MIMIC-III, a freely accessible critical care database Sci Data 3 160035 2016 27219127 10.1038/sdata.2016.35 PMC4878278

[18] MetaMap: A tool for recognizing UMLS concepts in text. https://metamap.nlm.nih.gov

[19] Reátegui R Ratté S Comparison of MetaMap and cTAKES for entity extraction in clinical notes BMC Med Inform Decis Mak 18 74 2018 suppl 3 30255810 10.1186/s12911-018-0654-2 PMC6157281

[20] Wu ST Liu H Li D et al Unified Medical Language System term occurrences in clinical notes: A large-scale corpus analysis J Am Med Inform Assoc 19 e1 e149 e156 2012 22493050 10.1136/amiajnl-2011-000744 PMC3392861

[21] Mauri M, Elli T, Caviglia G, et al: RAWGraphs: A visualisation platform to create open outputs. Presented at the 12 Biannual Conference of the Italian SIGCHI Chapter, Cagliari, Italy, September 18-20, 2017

[22] Choueiri TK Escudier B Powles T et al Cabozantinib versus everolimus in advanced renal-cell carcinoma N Engl J Med 373 1814 1823 2015 26406150 10.1056/NEJMoa1510016 PMC5024539

[23] Hussain M Daignault-Newton S Twardowski PW et al Targeting androgen receptor and DNA repair in metastatic castration-resistant prostate cancer: Results from NCI 9012 J Clin Oncol 36 991 999 2018 29261439 10.1200/JCO.2017.75.7310 PMC6075827

[24] Mascarenhas JO Talpaz M Gupta V et al Primary analysis of a phase II open-label trial of INCB039110, a selective JAK1 inhibitor, in patients with myelofibrosis Haematologica 102 327 335 2017 27789678 10.3324/haematol.2016.151126 PMC5286940

[25] Ernecoff NC Wessell KL Hanson LC et al Electronic health record phenotypes for identifying patients with late-stage disease: A method for research and clinical application J Gen Intern Med 34 2818 2823 2019 31396813 10.1007/s11606-019-05219-9 PMC6854193

[26] Bender D, Sartipi K: HL7 FHIR: An Agile and RESTful approach to healthcare information exchange. Presented at the 26 IEEE International Symposium on Computer-Based Medical Systems, Porto, Portugal, June 20-22, 2013

[27] National Institutes of Health Office of Data Science Strategy: Fast Healthcare Interoperability Resources (FHIR) standard. https://datascience.nih.gov/foa/fast-healthcare-interoperability-resources-fhir-standard

[28] Visweswaran S Becich MJ D’Itri VS et al Accrual to Clinical Trials (ACT): A Clinical and Translational Science Award Consortium Network JAMIA Open 1 147 152 2018 30474072 10.1093/jamiaopen/ooy033 PMC6241502

[29] Fleurence RL Curtis LH Califf RM et al Launching PCORnet, a national patient-centered clinical research network J Am Med Inform Assoc 21 578 582 2014 24821743 10.1136/amiajnl-2014-002747 PMC4078292

[30] Zhang Y, Staley SA, Tucker K, et al: Malignant Brenner tumor of the ovary: Case series and review of treatment strategies. Gynecol Oncol Rep 28:29-32, 2019 10.1016/j.gore.2019.02.003 PMC6378317 30815527

[31] Tsao PA, Estes JP, Griggs JJ, et al: Cardiovascular and metabolic toxicity of abiraterone in castration-resistant prostate cancer: Post-marketing experience. Clin Genitourin Cancer 17:e592-e601, 2019 10.1016/j.clgc.2019.03.001 31023520

[32] Siontis BL, Zhao L, Leja M, et al: Primary cardiac sarcoma: A rare, aggressive malignancy with a high propensity for brain metastases. Sarcoma 2019:1960593, 2019 10.1155/2019/1960593 PMC6431449 30962762

[33] Lazo de la Vega L, Samaha MC, Hu K, et al: Multiclonality and marked branched evolution of low-grade endometrioid endometrial carcinoma. Mol Cancer Res 17:731-740, 2019 10.1158/1541-7786.MCR-18-1178 30610106

[34] Shankar PR, Barkmeier D, Hadjiiski L, et al: A pictorial review of bladder cancer nodal metastases. Transl Androl Urol 7:804-813, 2018 10.21037/tau.2018.08.25 PMC6212631 30456183

[35] Hertz DL, Glatz A, Pasternak AL, et al: Integration of germline pharmacogenetics into a tumor sequencing program. JCO Precis Oncol 10.1200/PO.18.00011 10.1200/PO.18.00011 PMC7434089 32832831

[36] Morag Y, Yablon C, Brigido MK, et al: Imaging appearance of well-differentiated liposarcomas with myxoid stroma. Skeletal Radiol 47:1371-1382, 2018 10.1007/s00256-018-2940-6 29663025

[37] Aslam A, Mendiratta-Lala M, Curci ME, et al: Role of pelvic CT during surveillance of patients with resected biliary tract cancer. Abdom Radiol (NY) 45:116-122, 2020 10.1007/s00261-019-02159-0 31385009

[38] Chappell G, Geer M, Gatza E, et al: Maintenance sorafenib in FLT3-ITD AML following allogeneic HCT favorably impacts relapse and overall survival. Bone Marrow Transplant 54:1518-1520, 2019 10.1038/s41409-019-0493-5 30809038

[39] Manohar PM, Beesley LJ, Bellile EL, et al: Prognostic value of FDG-PET/CT metabolic parameters in metastatic radioiodine-refractory differentiated thyroid cancer. Clin Nucl Med 43:641-647, 2018 10.1097/RLU.0000000000002193 PMC6076335 30015659

[40] MetaMap: List of semantic types. https://metamap.nlm.nih.gov/Docs/SemanticTypes_2018AB.txt

